# Supplementary material for: Evolution of the quantum Hall bulk spectrum into chiral edge states
Source: Nat Commun. 2018 Sep 12;9:3692. doi: 10.1038/s41467-018-06025-3 (PMC6135798; doi:10.1038/s41467-018-06025-3)
Supplement: Supplementary file 1 — Supplementary Information [file 41467_2018_6025_MOESM1_ESM.pdf]

Evolution of the quantum Hall bulk spectrum into chiral edge states

Patlatiuk and Scheller et al.

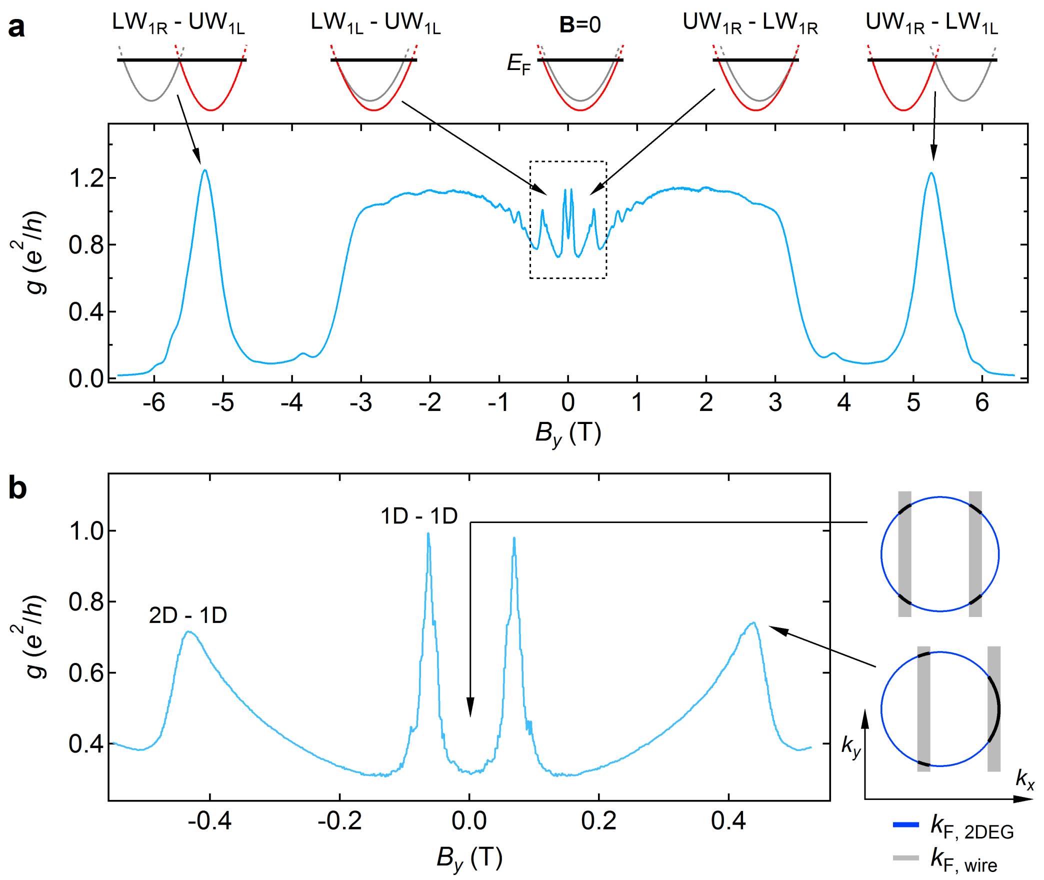

Supplementary Figure 1. Momentum resolved tunneling spectroscopy. a, The zero bias tunneling conductance as a function of in-plane magnetic field B_y peaks whenever upper and lower wire Fermi-points overlap, indicated by black arrows. UW (LW) dispersions are shown in red (gray) for different magnetic fields. Black solid bars indicate the Fermi energy. **b,** Zoom - in of dashed rectangle in **a,** showing symmetric wire transitions and asymmetric 2DEG-wire transitions. Sketches schematically depict the wave function overlap (black) in momentum space at the Fermi energy between 2DEG (dark blue circle) and lower wire modes (gray lines).

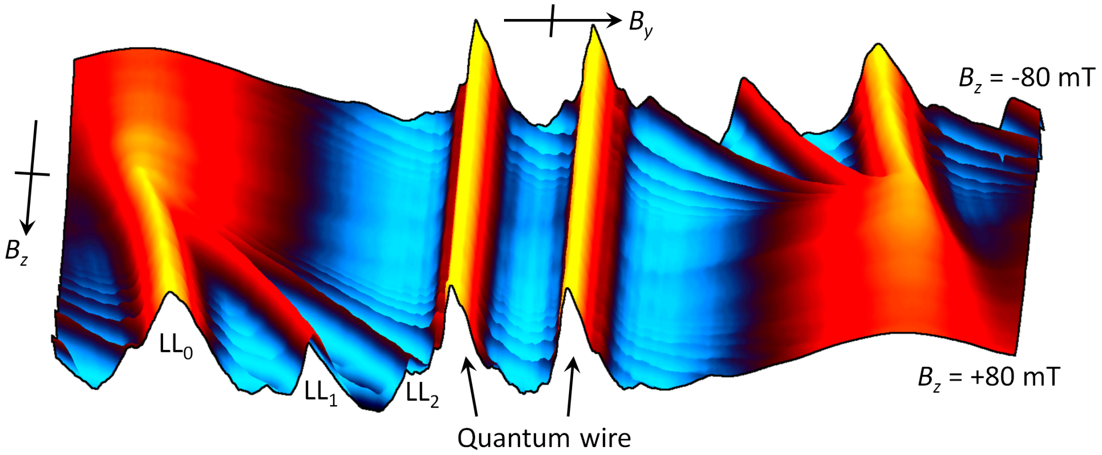

Supplementary Figure 2. Landau level formation. a, Measured tunneling conductance as a function of magnetic fields B_y and B_z. The 2DEG to quantum wire transition is observed to fan out into individual resonances in presence of a small perpendicular magnetic field, signaling the formation of discrete edge states. The underlaying Landau levels, LL_0, LL_1, and LL_2 for the first three edge states are indicated.

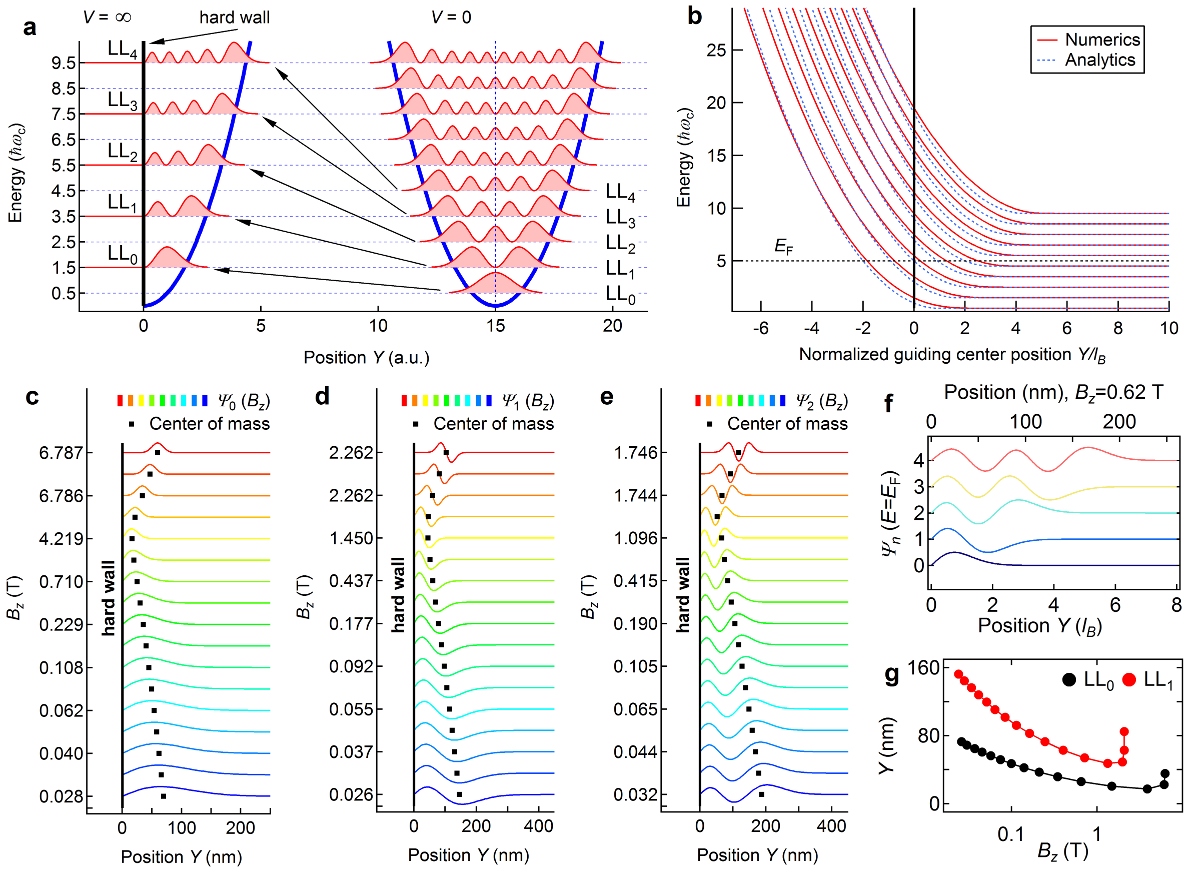

Supplementary Figure 3. Numerical solutions for hard wall confined Landau levels. **a**, LL wave functions squared (red) in the bulk, and for the case when the GC is placed exactly at the edge of the sample (bold black). Blue parabolas denote the magnetic field confinement. **b**, Numerical (red) and analytical solution from the main text (blue) for the energy evolution of LLs as a function of GC position close to the hard wall. **c-e** Evolution of the electron wave function at the Fermi energy with perpendicular magnetic field B_z of LL_0, LL_1, and LL_3, respectively. Hybridization with the upper quantum wire (solution of the Schrödinger equation for same confinement problem) would lead to an additional node in the wave function, as illustrated in Fig.1**e** of the main manuscript. **f** Wave function for all five LLs at $E_F = 5\hbar\omega_c$ ($B_z \approx 0.62\,\mathrm{T}$ for the present sample). **g** Center of mass (CM) B_z evolution for the lowest two LLs.

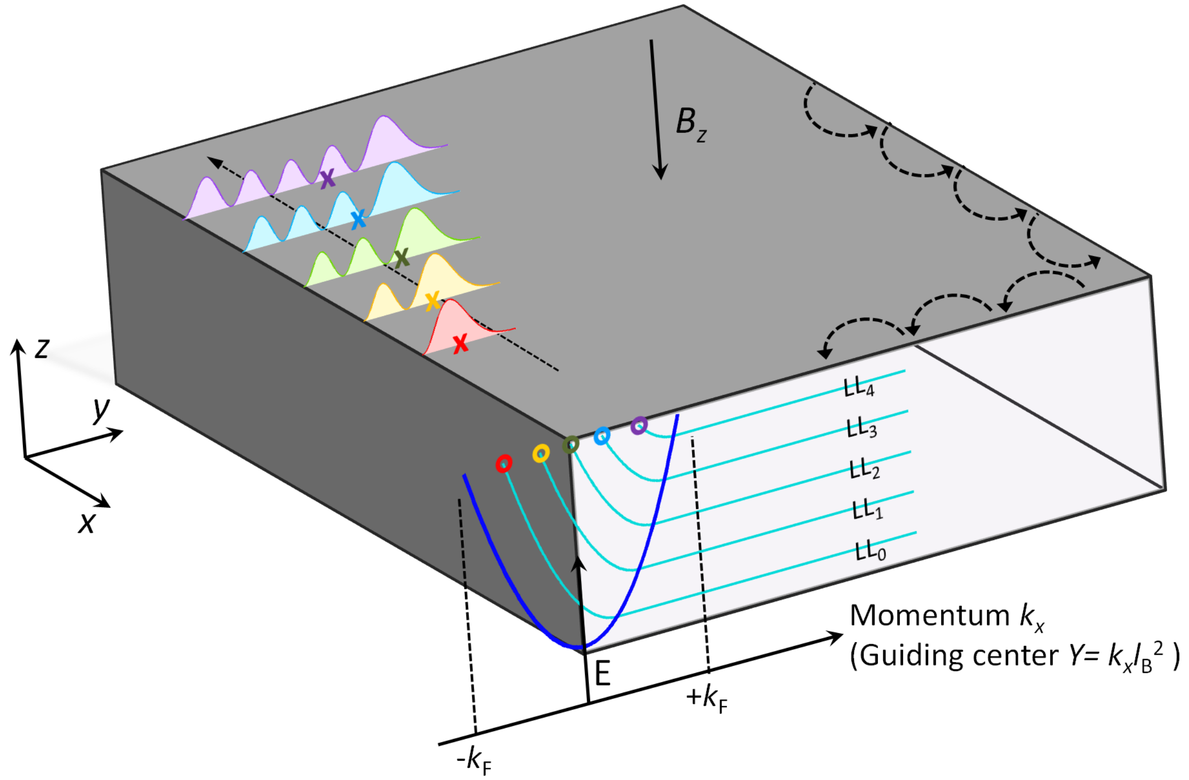

Supplementary Figure 4. Edge states at the hard wall. Schematic representation of the sample showing the Landau level dispersions in light blue for states propagating along the negative x-direction at the left sample edge (see black coordinate system). The edge states are formed at the intersection with the Fermi level. The corresponding momenta (GC positions) are indicated by colored circles. The wave functions squared are also shown and reside entirely within the sample. The hard wall confinement separates the GC positions of edge states (colored circles) from their wave function CM positions (colored crosses) at the sample edge. Classical cyclotron orbits are shown as dashed semi circles.

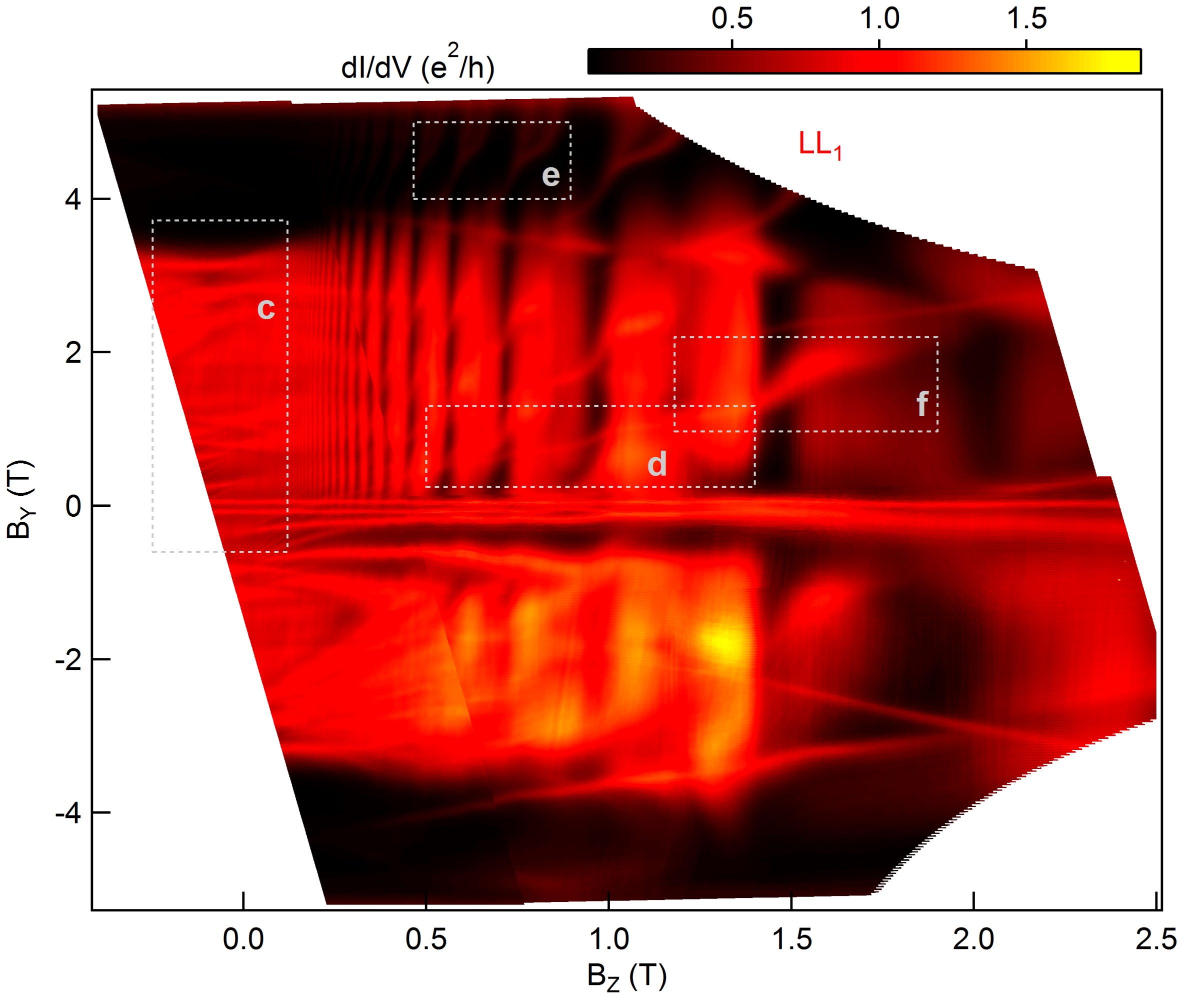

Supplementary Figure 5. Raw data of Fig.3a from the main text. Differential conductance measured using standard lock-in technique as a function of perpendicular (B_z) and in-plane (B_y) magnetic fields. Small tilt corrects the slight misalignment of the sample with respect to the magnet axes. The data in Fig. 3a of the main manuscript was differentiated twice with respect to B_y compared to the set shown here.

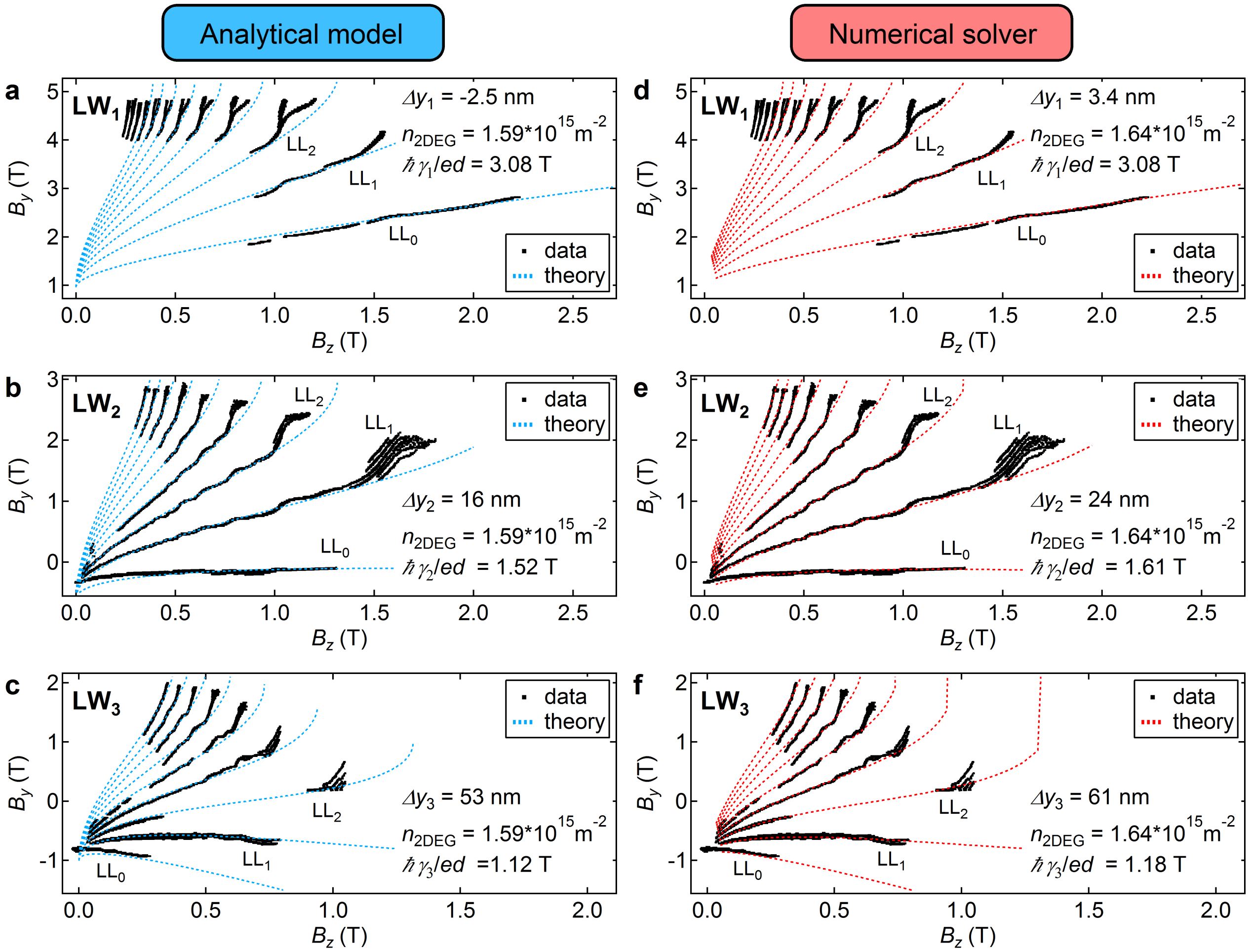

Supplementary Figure 6. Landau Level tunneling to different quantum wire modes. Extracted resonances (black) for tunneling between Landau levels and the lowest quantum wire modes LW_1, LW_2, and LW_3 are shown in the top, middle and bottom panels, respectively. Superimposed are theoretical predictions using the analytical model from the main article (blue, panels **a-c**) as well as numerical calculations (red, panels **d-f**) performed with a one dimensional single particle Schrödinger solver.

Supplementary Note 1. Tunneling spectroscopy at zero perpendicular magnetic field.

Tunneling spectroscopy in cleaved edge overgrown quantum wires [1, 2] allowed for extraction of the quantum wire dispersion relation [3] and has revealed a number of exciting phenomena such as spin charge separation [4], charge fractionalization[5], and interaction effects beyond the Luttinger liquid picture [6]. The CEO wires are ballistic [7], exhibit clear conductance quantization [8], and show a number of other Luttinger liquid signatures [9], such as e.g. ubiquitous power laws [10, 11] or interaction induced helical order of electronic and nuclear spin system [12–14]. As such CEO wires can serve as ideal momentum selective tunnel probes for the investigation of 1D systems such as the edge states in the integer quantum Hall regime.

In the tunneling spectroscopy configuration, shown in Fig. 1**c** of the main article, a surface gate depletes the two dimensional electron gas (2DEG) beneath and the adjacent upper wire (UW), while leaving a single conducting mode in the lower wire (LW), located beneath the UW. A magnetic field B_y, applied perpendicular to the plane spanned by the UW and LW (i.e. in the plane of the 2DEG), introduces a momentum kick to tunneling electrons, thereby shifting UW and LW wire dispersions with respect to each other. Supplementary Fig. 1 shows the zero bias tunneling conductance as a function of B_y, which peaks whenever upper and lower wire Fermi-points overlap (black arrows). Four such peaks are seen, corresponding to tunneling between counter-propagating electrons in the first UW and LW mode (either left or right movers) at $B_y = \pm 5.25\,\mathrm{T}$ or tunneling between co-propagating electrons at $B_y = \pm 65\,\mathrm{mT}$, in agreement with earlier experiments [3, 5]. A zoom-in for the low magnetic field region is shown in Supplementary Figure 1**b**. In addition to the co-propagating wire-wire transitions, appearing as symmetric peaks (symmetric with respect to their peak position) around $B_y = \pm 65\,\mathrm{mT}$ there is a second set of asymmetric peaks at $B_y = \pm 0.4\,\mathrm{T}$ with a sharp cut-off towards larger fields and a long tail at small field $|B_y| < 0.4\,\mathrm{T}$. While those peaks were previously assigned to tunneling among higher modes in the upper and lower quantum wire [5], the asymmetric peak shape suggests yet another origin. The peak shape may be qualitatively explained with a 2DEG to quantum wire transition, where the zero bias tunneling intensity is given by the overlap (black segments in the schematics of Supplementary Fig. 1**b**) in momentum space of a Fermi circle from the 2DEG and a pair of

vertical lines from the quantum wire with well defined momentum $\pm k_{\rm F}$ along the x-direction only, the direction of free propagation.

This scenario is confirmed in Supplementary Fig. 2 and Fig. 2 of the main article, where the 2DEG to quantum wire transition is observed to fan out into individual lines (Landau level formation) in presence of a perpendicular magnetic field B_z, while transitions among quantum wires remain unaffected.

Supplementary Note 2. Landau levels in Landau-gauge: Introduction and terminology.

In order to introduce the proper terminology the simple quantum mechanical problem of a free electron in the x-y plane, subject to a strong perpendicular magnetic field B_z, is briefly reviewed. We choose Landau gauge with the vector potential $\mathbf{A} = (-B_z \cdot y, 0, 0)$ giving a magnetic field $\mathbf{B} = \nabla \times \mathbf{A} = (0, 0, -B_z)$, and the sample edge runs along the x-direction. The Hamiltonian of the system reads

$$\hat{H} = \frac{(\mathbf{p} - e\mathbf{A})^2}{2m^*} = \frac{\hat{p}_y^2}{2m^*} + \frac{1}{2}m^* \left(\hat{p}_x + eB_z y\right)^2 \qquad (1)$$

Here, $\mathbf{p}$ denotes the momentum operator with components $\hat{p}_x$ and $\hat{p}_y$, e is the elementary charge, and m^* the effective electron mass. Since $\hat{H}$ and $\hat{p}_x$ commute, $\hat{p}_x$ can be replaced by its eigenvalue $\hbar k_x$:

$$\hat{H} = \frac{\hat{p}_y^2}{2m^*} + \frac{1}{2}m^*\omega_{\rm c}^2 \left(y + \underbrace{k_x \cdot l_B^2}_{Y}\right)^2 \quad , \qquad (2)$$

where we have introduced the cyclotron frequency $\omega_{\rm c} = eB_z/m^*$, the magnetic length $l_B = \sqrt{\hbar/eB_z}$, and $\hbar$ is the reduced Planck constant. The system consists of standard quantum harmonic oscillators, shifted in y-direction by the guiding center (GC) position Y, giving rise to the well known energy spectrum $E_n = \hbar\omega_{\rm c}(n + 1/2)$, and hermite-gaussian wave functions Ψ_n, see Supplementary Figure 3. We note that the GC Y intimately links the effective momentum along the x-direction to the y-displacement from the edge through $k_x = Y/l_B^2$, which is key to the momentum resolved LL spectroscopy performed here. This can simply be understood in terms of the classical Lorentz force: Upon e.g. moving an

electron closer to the edge by an amount Δy it is subject to the Lorentz force $F = e\frac{\Delta y}{\Delta t}B_z = \frac{\hbar \Delta k_x}{\Delta t}$ and hence experiences a momentum kick $\Delta k_x = \Delta y \frac{eB_z}{\hbar} = \Delta y / l_B^2$.

Upon approaching the edge with the GC position Y, the hard wall introduces a sharp cut-off to the parabolic magnetic confinement. Placing the GC exactly at the hard wall, one obtains a particularly simple case, where the odd bulk LLs provide the exact solution for the "half-parabola" confinement problem (due to their node at the GC position), i.e. $\mathrm{LL}_n \rightarrow \mathrm{LL}_{2n+1}$ at the edge, see Supplementary Figure 3**a**.

Supplementary Note 3. Numerical solutions for Landau levels at sharp edges

In order to calculate the continuous evolution of LLs and their wave functions, we numerically solve the Schrödinger equation using Numerov's method for the parabolic confinement (from the magnetic field), partially chopped off by the hard wall (depending on the GC position).

We normalize the energy by substituting $\hbar\omega_\mathrm{c} = 1$ and the spatial coordinate by setting $l_B = 1$. This transforms the 1D Schrödinger equation into a unit-less form, which is then solved in the real space interval $y = [0, 25]l_B$ (within the physical sample) for different GC positions $Y = [-7, 10]l_B$ (Supplementary Figure 3**b**). For a physical solution the wave function has to be zero at the hard wall and far inside the sample (exponential damping due to the magnetic field confinement), hence $\Psi(0) = 0$ and $\Psi(25) = 0$. For each GC position the energy is changed iteratively until a solution is found that satisfies the right boundary condition $\Psi(25) = 0$ with sufficient precision ($\Psi(25) \ll 1$ for a normalized wave function). However, in order to obtain faster convergence, a more complicated method is implemented. Here we set $\Psi(0) = \Psi(25) = 0$ and calculate the wave function from both sides. This requires stitching together of the two halves such that the total wave function remains continuously differentiable. The procedure allows us to calculate the evolution of energy and wave functions versus the GC position (Supplementary Figure 3**b-g**).

Supplementary Figure 3**b** shows the numerically obtained energy evolution for the first 10 LLs (red) along with predictions from the analytical model (blue) described in the main text. While the analytical model generally agrees very well with the numerics, there are some discrepancies at low B-field (i.e. when the Fermi energy E_F is much larger than the cyclotron energy $E_\mathrm{c} = \hbar\omega_\mathrm{c}$) and close to depletion of individual LLs in the bulk.

Since all spectroscopy experiments performed here are done at zero bias, tunneling only involves electronic states close to E_{F}. Hence, we are interested in the GC position Y_n where LL_n intersects with the Fermi energy (Supplementary Figure 3**b**). Tracking these intersections as a function of B_z allows one to predict the real-space evolution of integer quantum hall edge states, shown in Supplementary Figure 3**c-e** on a non-linear scale for LL_0, LL_1, and LL_2, respectively. Since the total width σ_n of the $\mathrm{n^{th}}$ edge states scales with the magnetic length, $\sigma_n \approx 2l_B\sqrt{2n+1}$, LLs are slowly compressed towards the hard wall upon increasing B_z. However, once the respective filling factor is reached the corresponding LL moves abruptly back into the bulk and is magnetically depopulated (lifted above E_{F}). This is clearly visible in Supplementary Figure 3**g**, where the center of mass (CM) position (black dots in Supplementary Figure 3**c-e**) for LL_0 and LL_1 is shown as a function of B_z.

Comparing the wave functions of different edge states for $E_{\mathrm{F}} = 5\hbar\omega_{\mathrm{c}}$ (Supplementary Figure 3**f**), we note their spatial overlap, while corresponding GCs in Supplementary Figure 3**b** are well separated. This suggests massively improved measurement sensitivity by means of momentum resolved spectroscopy compared to real-space imaging of edge states using local probes.

The results of the numerical simulations are summarized in the schematic 3D representation of the sample shown in Supplementary Figure 4. The Landau level dispersions are plotted on the sample front side and describe propagation along the sample edge (x-direction, see coordinate system). The GC positions for the different edge states (intersection of the dispersion with the Fermi energy) are indicated with colored circles and reside outside the physical sample for edge states associated with Landau levels of low orbital index. The wave functions, on the other hand, are contained within the sample for all edge states, leading to a separation of GC position (colored circles) and edge state CM position (colored crosses) in samples with hard wall confinement.

Supplementary Note 4. Comparison of theory and experiment

Here we compare experimental data for tunneling to the first three lower wire modes (Fig. 3 in the main article) to both, theoretical predictions using the numerical Schrödinger solver described in detail in the previous section, and predictions using the analytical model introduced in the main text.

While calculating the LL dispersion in Supplementary Figure 3**b** requires only one free parameter, namely the 2DEG density n_{2DEG}, two additional parameters have to be introduced in order to compare theoretical predictions and experimental data. On one hand, each quantum wire mode has its specific density and consequently differing Fermi wave-vector. The corresponding momentum mismatch of quantum wire and 2DEG (i.e. the tunneling condition at $B_z = 0$) is compensated with the in-plane magnetic field B_y, thus vertically shifting Landau fans in the $B_y - B_z$ measurement plane for different wire modes (see Fig. 2 and Fig. 3 in the main article). On the other hand, the wire wave functions are of finite width and hence the mode-specific CM position y_n is slightly displaced from the hard wall towards the bulk of the sample, while the LL dispersions in Supplementary Figure 3**b** are calculated with respect to the sample edge. The displacement gives raise to an additional momentum kick $\hbar k = e y_n B_z$, linear in B_z, which for small y_n appears like a rotation of fan structures around the origin $\mathbf{B} = (0, B_y, B_z) = 0$.

A comparison between theoretical predictions and experimental data is shown in Supplementary Figure 6. While both, numerical solutions and analytical model agree well with the measured data, there are some discrepancies at low B_z, in particular for the analytical case. In addition, both theoretical models do not take the spin degree of freedom into account and hence do not reproduce the spin splitting, seen for large in-plane magnetic field $B_y \gtrsim 4.5\,\mathrm{T}$ in Supplementary Figure 6**a,d** and Fig. 3**e** in the main text.

Investigating the fitting parameters, we note that the 2DEG density $n \approx 1.6 \cdot 10^{15}\mathrm{m}^{-2}$ turns out to be the same for all data sets (for both, analytical and numerical model). This strongly supports the theoretical models used here, since the 2DEG density should not depend on the specific wire mode used as a spectrometer for imaging of the quantum hall edge states.

Besides investigation of Landau levels, the fits in Supplementary Figure 6 allow us to reconstruct the quantum wire mode structure, i.e. determine their CM position. For the tri-

angular confinement potential at the sample edge, the quantum wire modes rapidly decrease in sub-band spacing with increasing mode index, while their width (extension of the wave function) is expected to significantly increase. Indeed, the CM position for LW_1 is located very close to the sample edge, whereas the position for the second and third lower wire mode are displaced much more from the hard wall, see fitting parameters in Supplementary Figure 6. We note that for the analytical model Δy_1 turns out negative, which is unphysical for a sample with hard wall, i.e. this model effectively describes a situation with slightly weaker confinement.

Supplementary References

[1] Pfeiffer, L. *et al.* Cleaved edge overgrowth for quantum wire fabrication. *J. Cryst. Growth.* **127**, 849 (1993).

[2] Wegscheider, W. *et al.* High-mobility transport along single quasi-1d quantum wires formed by cleaved edge overgrowth. *Solid State Electron.* **37**, 547 (1994).

[3] Auslaender, O. M. *et al.* Tunneling spectroscopy of the elementary excitations on a one-dimensional wire. *Science* **295**, 825 (2002).

[4] Auslaender, O. M. *et al.* Spin-charge separation and localization in one dimension. *Science* **308**, 88 (2005).

[5] Steinberg, H. *et al.* Charge fractionalization in quantum wires. *Nat. Phys.* **4**, 116 (2008).

[6] Barak, G. *et al.* Interacting electrons in one dimension beyond the luttinger-liquid limit. *Nat. Phys.* **6**, 489 (2010).

[7] de Picciotto, R., Störmer, H. L., Pfeiffer, L. N., Baldwin, K. W. & West, K. W. Four-terminal resistance of a ballistic quantum wire. *Nature* **411**, 51 (2001).

[8] Yacoby, A. *et al.* Nonuniversal conductance quantization in quantum wires. *Phys. Rev. Lett.* **77**, 4612 (1996).

[9] Auslaender, O. M. *et al.* Experimental evidence for resonant tunneling in a luttinger liquid. *Phys. Rev. Lett.* **84**, 1764 (2000).

[10] Tserkovnyak, Y., Halperin, B. I., Auslaender, O. M. & Yacoby, A. Interference and zero-bias

anomaly in tunneling between luttinger-liquid wires. *Phys. Rev. B* **68**, 125312 (2003).

[11] Tserkovnyak, Y., Halperin, B. I., Auslaender, O. M. & Yacoby, A. Finite-size effects in tunneling between parallel quantum wires. *Phys. Rev. Lett.* **89**, 136805 (2002).

[12] Scheller, C. P. *et al.* Possible evidence for helical nuclear spin order in gaas quantum wires. *Phys. Rev. Lett.* **112**, 066801 (2014).

[13] Braunecker, B., Simon, P. & Loss, D. Nuclear magnetism and electron order in interacting one-dimensional conductors. *Phys. Rev. B* **80**, 165119 (2009).

[14] Meng, T. & Loss, D. Helical nuclear spin order in two-subband quantum wires. *Phys. Rev. B* **87**, 235427 (2013).
